# Experimental Investigation on Ultra-Thin Vapor Chamber with Composite Wick for Electronics Thermal Management

**DOI:** 10.3390/mi15050627

**Published:** 2024-05-07

**Authors:** Shiwei Zhang, Haoyi Huang, Jingjing Bai, Caiman Yan, Huarong Qiu, Yong Tang, Fangqiong Luo

**Affiliations:** 1 Intelligent Manufacturing Engineering Laboratory of Functional Structure and Device in Guangdong, School of Mechanical and Automotive Engineering, South China University of Technology, Guangzhou 510640, China; swzhang@scut.edu.cn (S.Z.); howii1999@163.com (H.H.); me_chesbai@mail.scut.edu.cn (J.B.); chamenyan@163.com (C.Y.); mehrqiu@mail.scut.edu.cn (H.Q.); ytang@scut.edu.cn (Y.T.); 2 SCUT-Zhuhai Institute of Modern Industrial Innovation, Zhuhai 519175, China; 3 School of Mechanical and Control Engineering, Shenzhen University, Shenzhen 518000, China

**Keywords:** ultra-thin vapor chamber, composite mesh wick, phase change, heat transfer performance

## Abstract

Ultra-thin vapor chambers (UTVCs) are widely used to cool high-power electronics due to their excellent thermal conductivity. In this study, a UTVC of 82 mm × 58 mm × 0.39 mm with composite wick was prepared. The composite wick is composed of two layers of copper mesh and multiple spiral-woven meshes (SWMs), and the composite wick was applied in UTVC to improve liquid replenishment performance and temperature uniformity. Furthermore, the thermal performance of UTVCs with different support column diameters, filling ratios (FRs), and SWM structures was experimentally studied. The results found that the equivalent thermal conductivity (ETC) decreases as the diameter of the support column increases; the UTVC with 0.5 mm support column diameter has the highest ETC, at 3473 W/(m·K). Then, the effect of FR on the heat transfer performance of UTVCs with SWM numbers of 0, 1, 2, and 3 (0 SWMs, 1 SWM, 2 SWMs, 3 SWMs) is consistent, the 30% FR UTVC with 3 SWMs having the highest ETC, at 3837 W/(m·K). Finally, the increased number of SWMs can significantly improve the ultimate power of the UTVCs, the UTVC with 3 SWMs having the highest ultimate power, at 26 W. The above experimental studies indicate that the designed and manufactured UTVCs have great potential advantages in thermal dissipation for electronics.

## 1. Introduction

With the continuous advancement of microelectronics technology, various electronics are gradually developing in the direction of miniaturization, integration, and high performance [1,2,3]. The limited space of electronics makes it difficult to efficiently export heat, resulting in a significant temperature rise in electronics. Related studies have shown that the reliability and service life of electronics will decrease with high temperature and even lead to electronics failure [4,5]. Therefore, developing new and efficient thermal management technologies to achieve effective heat dissipation in electronics is an urgent issue that needs to be addressed [6,7,8].

Phase change heat transfer devices utilize the latent heat of phase changes of the working fluid to carry away heat; this is the most promising thermal management method to solve the heat dissipation problem in electronics [9,10,11]. Due to the difficulty in applying conventional-sized phase change heat transfer devices in miniaturized electronics, currently, ultra-thin phase change heat transfer devices are the focus of industry [12]. Commonly used ultra-thin phase change heat transfer devices mainly include ultra-thin heat pipes (UTHPs) and ultra-thin vapor chambers (UTVCs). UTHPs are usually made by first manufacturing round heat pipes with ultra-thin walls, and then processing them by heating and flattening. There has been much research on UTHPs [13,14]. Zhou et al. [15] developed an innovative UTHP with a double-hole spiral-woven mesh (SWM), the results show that the thickness and maximum heat transfer performance of the UTHP are 1.1 mm and 24 W, respectively. Sanhan et al. [16] used the finite element method to predict and simulate the heat transfer performance of a cylindrical heat pipe with a total length of 200 mm and flattened thicknesses of 2, 3, and 4 mm; the optimal design thickness of the UTHP was optimized is 2.5 mm. Zhou et al. [17] designed three different SWM structures with a thickness of 0.4 mm to study the effect of the wick on the heat transfer performance of UTHPs. Further increasing the number of SWM wires did not significantly improve the heat transfer performance of UTHPs, and the maximum heat transfer performance of the UTHPs was 4.25 W, 5 W, and 5.25 W, respectively. Li et al. [18] proposed three composite wick structures with a thickness of 1 mm, namely single-arched sintered groove wicks (SSGWs), double-sided arched sintered groove wicks (BSGWs), and mesh groove wicks (MGWs) to improve thermal transfer performance of UTHPs; at the optimal filling ratio (FR), the corresponding thermal transfer performance are 12 W, 13 W, and 14 W, respectively. However, due to the difficult preparation process of ultra-thin-walled copper tubes, the thickness of UTHPs after flattening is mostly 1 mm, and the performance deteriorates significantly when the thickness is further reduced. Therefore, UTHPs are generally unable to meet the cooling needs of tiny electronics with the advent of the 5G era.

In particular, the outer dimensions of UTVCs can be adjusted according to actual heat dissipation demands; simultaneously, they have advantages such as excellent thermal conductivity, large heat transfer area, and good temperature uniformity performance [19]. Scholars have conducted extensive research on the design, manufacturing, and performance of UTVCs [20,21,22]. Huang et al. [23] adopted four SWMs and a copper mesh as the wick to create a gas–liquid coplanar UTVC with a thickness of 0.5 mm, and the ultimate power of the UTVC was 7.58 W when placed horizontally. Lim et al. [24] used laser processing to prepare a fan-shaped micro-groove wick with a depth of 0.3 mm and packaged it into a copper-based UTVC with a size of 56 mm × 8 mm × 1.5 mm; the results show that its thermal resistance is 5.45 °C/W when the power is 8 W. Ding et al. [25] manufactured a titanium-based UTVC with a size of 30 mm × 30 mm × 0.6 mm, and experimental results showed that the maximum equivalent thermal conductivity (ETC) of the UTVC was 350 W/(m·K). Shi et al. [26] developed a UTVC with a size of 80 mm × 50 mm × 0.65 mm, copper mesh with a diameter of 76 μm was used as wick, and it was found that the UTVC had better heat transfer performance when the input power was in the range of 7.1 W to 13.7 W. Xu et al. [27] sintered four layers of copper mesh as a wick to prepare a UTVC with an effective size of 35 mm × 35 mm × 0.62 mm, and the maximum heat flux density that could be transmitted along the thickness direction reached 425 W/cm^2^. Chen et al. [28] prepared a 0.3 mm-thick UTVC with radial multi-arterial reentrant microchannels by sintering copper powder with a particle size of 50–75 μm as the wick.

As previously indicated, existing UTVCs are mainly groove type, mesh type, or powder-sintering type. However, a grooved UTVC is not suitable for cooling electronics with directional requirements because the grooved wick has poor antigravity properties [15]. The thinner powder-sintered layer results in a lower yield and poor heat transfer performance of UTVC [29]. Moreover, most of the above studies are about UTVCs with homogeneous wicks; however, composite wicks can typically combine the advantages of each component to increase the overall permeability and capillary force of the wick [30]. Many experiments have shown that the heat transfer performance of heat pipes can be enhanced by designing and optimizing the composite wick structure of the heat pipe [31,32,33]. In short, UTVCs have been widely studied, but there are few reports on UTVCs with thicknesses below 0.4 mm. To meet the ultra-thin heat dissipation requirements of electronics, the thickness and heat transfer performance of UTVCs still need further improvement, reasonable design and optimization of the structure of the composite wick inside UTVCs are expected to enhance its liquid replenishment performance and heat transfer performance.

In this work, a UTVC of 82 mm × 58 mm × 0.39 mm with a composite wick is prepared for the thermal dissipation of electronics. The composite wick is composed of two layers of copper mesh and multiple SWMs, and a chemical oxidation treatment of SWM is carried out to enhance its wettability. Wet etching is used to etch the support columns on the top shell to obtain a larger steam space. Then, secondary degassing of the UTVC is performed to promote better vacuum and heat transfer performance. Finally, the effects of the support column diameter, FRs, and wick structures on the heat transfer performance of UTVCs are fully explored. This work will provide reference value for the design of the support column structure, FR selection, and the arrangement of the composite wick structure of UTVC, and also provide a research foundation for the future process development of UTVC.

## 2. Experimental Section

### 2.1. Design and Manufacturing of UTVC

**Figure 1:**
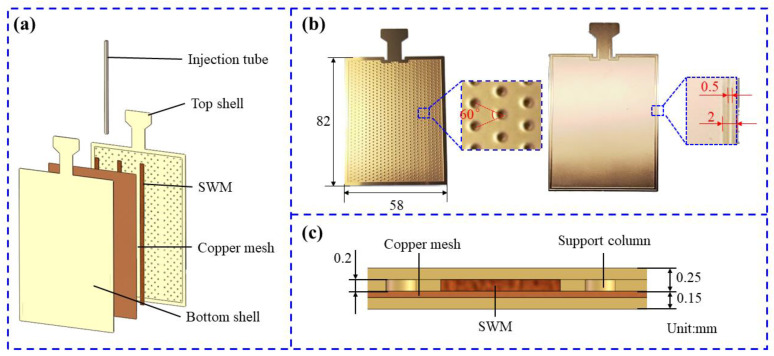
Structure and dimensions of UTVC: (**a**) Components of UTVC. (**b**) Structure and dimensions of the etched shell plates and pillars. (**c**) Cross-section dimensions of UTVC.

The structural components of the UTVC in this article are shown in Figure 1a, including the top shell, bottom shell, composite wick, and liquid injection tube. To meet the accuracy requirements for the UTVC’s shells and support columns which traditional cutting techniques cannot, wet etching is used to process top and bottom shells and support columns. The structural dimension parameters of the shells and support columns are shown in Figure 1b; the overall dimensions of the top and bottom shells are 58 mm × 82 mm, and the internal cavity size is 54 mm × 78 mm. Furthermore, the etching depth of the top shell is 0.2 mm, the support column spacing is also 2 mm, and the supporting columns are arranged in equilateral triangles to provide a steam channel running the width of the UTVC, thereby improving the overall temperature uniformity performance of UTVC. To provide flow space for the solder, reduce the thickness of the solder layer, and facilitate the positioning of the solder in the welding process, the etching depth of the bottom shell is set at 0.11 mm, and a 0.5 mm-wide groove at the welding edge is also incorporated into the design. Moreover, the top of the shells is designed with a “T”-type structure for stamping the liquid injection port. The cross-section of the UTVC is shown in Figure 1c, the top shell thickness of UTVC is 0.25 mm, the bottom shell thickness of UTVC is 0.15 mm, and the height of the support columns is 0.2 mm, to provide a 0.2 mm-high steam channel. The specific parameters of the designed UTVC are summarized in Table 1.

Moreover, the composite wick is composed of two layers of copper mesh and multiple SWMs, the thickness of the copper mesh is 0.06 mm, and the mesh size is 250. The SWM is woven from 24 strands of copper mesh, and each strand of copper mesh is composed of 6 copper wires with a diameter of 0.04 mm. Copper mesh is used to enhance the uniformity of temperature in the UTVC, and the SWM accelerates the reflux speed of the working fluid through unidirectional capillary force. The heat transfer performance of UTVC will be improved by combining the advantages of the two wicks.

**Figure 2:**
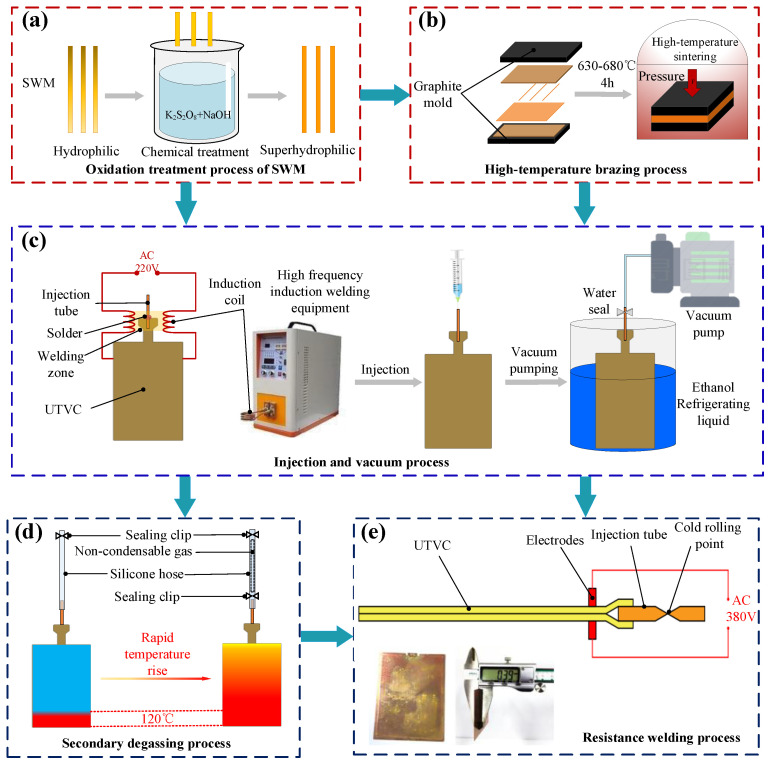
The manufacturing process of the UTVC. (**a**) Oxidation treatment process of SWM. (**b**) High-temperature brazing process. (**c**) Injection and vacuum process. (**d**) Secondary degassing process. (**e**) Resistance welding process.

The manufacturing process of the designed UTVC is shown in Figure 2a–e, including the oxidation treatment process of SWM, high-temperature brazing process, injection and vacuum process, secondary degassing process, and resistance welding process. First, an oxidation treatment method is used to improve the surface morphology of the SWMs, thus enhancing their hydrophilicity and capillary properties. Specifically, a mixed solution of 2 mol/L NaOH and 0.1 mol/L K_2_S_2_O_8_ is prepared; the dried SWM is oxidized in the solution for 60 min at room temperature, then washed with a large amount of deionized water, and finally dried in a vacuum drying oven, whereupon the oxidation treatment of SWM is completed, as shown in Figure 2a.

Then, the high-temperature brazing process is the most important step in the processing of UTVC, as it directly affects the sealing and yield of the UTVC and determines whether it can start normally. CB200 brand copper nickel solder paste is adopted to evenly spot-coat the solder groove of the bottom shell. The oxidized wick is assembled into the top shell, then the top and bottom shells are assembled and placed in the graphite mold. To control the weld thickness of the UTVC shells, a uniform pressure is applied to the graphite molds, and then the high-temperature brazing of UTVC is carried out in a bell sintering furnace. In the meantime, a mixture of 95% nitrogen and 5% hydrogen is continuously introduced into the bell furnace, which provides a protective atmosphere for the brazing process, prevents the oxidation of the wick at high temperatures, and also helps to reduce the organic matter and oxidation on the surface of the shells, thereby reducing the impurities in the UTVC after encapsulation. The heating process of the bell furnace is as follows: (1) The room temperature rises to 550 °C for 120 min; (2) rises from 550 °C to 650 °C, taking 60 min; (3) holds at 650 °C insulation for 60 min; (4) after natural cooling to room temperature, the bell furnace is opened and the UTVC is taken out. During this process, always introduce protective gas to prevent secondary oxidation of the samples. The high-temperature brazing process is shown in Figure 2b.

Next, injection and vacuum processes for UTVC are performed, as shown in Figure 2c. The first step in the injection and vacuum process is to weld the injection tube and the shell of the UTVC; a copper injection tube with an outer diameter of 2 mm and a wall thickness of 0.5 mm is adopted to weld to a UTVC shell through high-frequency induction welding. It is necessary to inspect the sealing of the UTVC after welding the injection tube, as the injection tube is connected to a vacuum pump through a silicone hose for vacuum pumping, and a vacuum degree of ≤0.5 Pa inside the UTVC is defined as well sealed. Next, since the melting point of deionized water is 0 °C under normal pressure, the injection process is carried out on the UTVC by the method of “injection first and then vacuum”; specifically, the working fluid is first injected into the UTVC and then quickly frozen, then the vacuuming step is carried out by keeping the temperature below 0 °C in ethanol refrigerating liquid.

Further, the secondary degassing process of the UTVC is carried out to improve the internal vacuum degree, as shown in Figure 2d. Due to the boiling point of the working medium decreasing with the increase of vacuum, the internal working medium will phase change in the process of vacuum pumping, and it is difficult to achieve a high vacuum degree inside the UTVC by the first vacuum pumping. At the same time, there will be a certain amount of non-condensable gas remaining in the UTVC, so it is necessary to remove the air through a secondary degassing process. During the secondary degassing process, the top end of the silicone hose is sealed with a sealing clip after the first degassing is completed, and a 120 °C heating device is adopted to heat the bottom of UTVC. The lower end of the silicone hose is sealed with a sealing clamp when the temperature at the injection port of the UTVC increases sharply, then the injection tube is cold-rolled and sealed with a hydraulic clamp.

Finally, the resistance welding sealing process of the UTVC is performed. The produced UTVC is prone to leakage if it is only sealed by cold-rolling after multiple gas–liquid cycles; therefore, the resistance welding process is used to remove the tail of the UTVC after secondary degassing, high-temperature hot pressing is carried out for the injection port, and then the top and bottom shells are melted and solidified to achieve a good sealing effect. The resistance welding process and the actual picture of the UTVC after encapsulation are shown in Figure 2e; the actual thickness of UTVC is 0.39 mm.

### 2.2. Heat Transfer Performance Test

**Figure 3:**
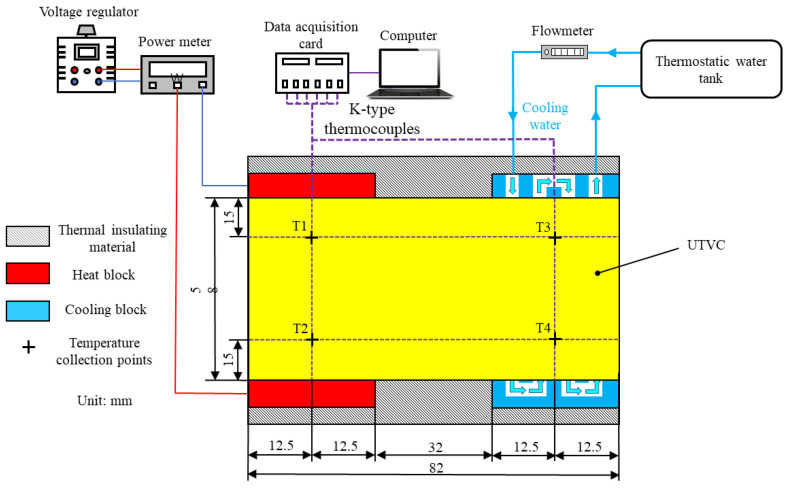
Steady-state thermal performance test system of UTVC.

A heat transfer performance test system was established to evaluate the heat transfer performance of the UTVC. The system is mainly composed of a heating module, cooling module, data acquisition module, and sample, as shown in Figure 3. The heating module includes an autotransformer, power monitor, and heating copper block, the heating area of the copper block is 58 mm × 25 mm, and in the experiment, the heating power starts at 5 W and increases by 5 W when the temperature reaches a stable value. A steady state is defined as the temperature change of each measurement point is less than 0.5 °C in 2 min. The cooling module includes a constant temperature water tank, flowmeter, and cooling block, the experiment was carried out under the condition of forced water cooling, the water temperature was controlled at 30 °C, and the flow rate was set to 2 L/min.

In addition, the heated copper block and water-cooling block are embedded in a synthetic stone plate, and all exposed surfaces of the sample are wrapped with insulation foam to minimize heat leakage, thus reducing the heat exchange between the experimental system and the external environment. Silicone grease (thermal conductivity > 4.85 W/(m·K)) is uniformly applied to the contact surface of the UTVC with the heating block and the water-cooling block to reduce the contact thermal resistance, increasing the accuracy of the experiment. Meanwhile, the four thermocouples $T_{1}-T_{4}$ are arranged on the upper surface of the UTVC; $T_{1}$ and $T_{2}$ are used to collect the temperature of the evaporation section, $T_{3}$ and $T_{4}$ are used to collect the temperature of the condensation section, and then temperature data measured by thermocouples $T_{1}-T_{4}$ are transferred to a computer via the data acquisition card for summarization.

### 2.3. Characterization of Wettability of Wick

**Figure 4:**
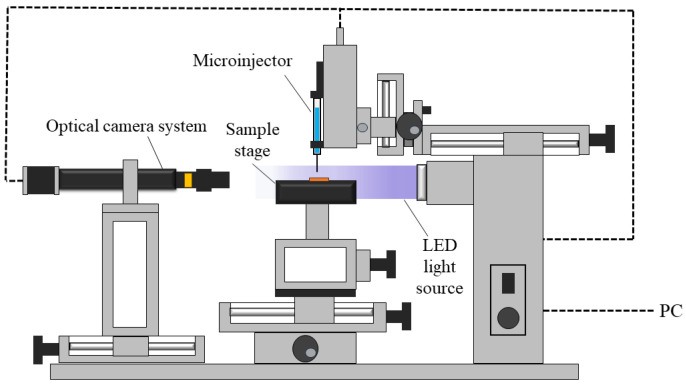
Contact angle measurement device.

To verify the effectiveness of chemical oxidation on SWM, a contact angle measurement device (OSA200-T, Ningbo NB Scientific Instruments, Ningbo, China) is used to test the wetting properties of the wick surface. The contact angle measurement device mainly includes a microinjector, a sample stage, an LED light source, and an optical camera system, as shown in Figure 4. The prepared sample is placed horizontally on the sample stage when testing, and the optical camera system is adjusted to focus and brighten the image. Deionized water is adopted as the test fluid, and its volume is controlled to 4 μL via a microinjector. Then adjust the sample table to move slowly upward until the fluid drops touch the sample surface. Finally, take photos through the optical shooting system and record the contact angle. The entire experimental process was carried out at a constant temperature of 25 °C.

### 2.4. Data Processing and Uncertainty

The ETC is used to evaluate the heat transfer performance of UTVC, and the derivation of the ETC is as follows.

First, the evaporating and condensing section average temperatures are calculated by the following equations:

(1) $$\left\{\begin{array}{l} {\bar{T}}_{e}=\frac{T_{1}+T_{2}}{2}\\ {\bar{T}}_{c}=\frac{T_{3}+T_{4}}{2} \end{array}\right.$$

where ${\bar{T}}_{e}$ represents the average temperature of the evaporating section; and ${\bar{T}}_{c}$ represents the average temperature of the condensing section.

The temperature difference ΔT between the evaporating and condensing sections is

(2) $$\Delta T={\bar{T}}_{e}-{\bar{T}}_{c}$$

Then, the total thermal resistance R of UTVC is expressed as

(3) $$R=\frac{\Delta T}{P}$$

where P represents the input power of the heating module.

Finally, the ETC $K_{eff}$ is calculated as

(4) $$\left\{\begin{array}{l} K_{eff}=\frac{L}{R\cdot A_{c}}\\ L=\frac{1}{2}L_{e}+L_{a}+\frac{1}{2}L_{c} \end{array}\right.$$

where $A_{c}$ represents the cross-sectional area of the UTVC; L represents effective length; $L_{e}$ represents the length of the evaporating section; $L_{a}$ represents the length of the adiabatic section; and $L_{c}$ represents the length of the condensing section.

To make the experimental data more reliable, the experimental uncertainty analysis is performed according to the standard error analysis method [34]. The uncertainties generated during the heat transfer performance testing of the UTVC are mainly the temperature error of thermocouples and the thermal loss error of the input thermal power. And K-type thermocouple has a temperature measurement accuracy of ±0.1 °C and a temperature acquisition card data has a reading accuracy of ±0.05 °C, then the temperature uncertainty is less than 0.2%. The uncertainties in the average temperatures of the evaporation and condensation sections are calculated as follows:

(5) $$\left\{\begin{array}{l} \frac{U_{{\bar{T}}_{e}}}{{\bar{T}}_{e}}=\sqrt{(\frac{U_{T_{1}}}{T_{1}+T_{2}}{)}^{2}+(\frac{U_{T_{2}}}{T_{1}+T_{2}}{)}^{2}}\\ \frac{U_{\bar{T}c}}{{\bar{T}}_{c}}=\sqrt{(\frac{U_{T_{3}}}{T_{3}+T_{4}}{)}^{2}+(\frac{U_{T_{4}}}{T_{3}+T_{4}}{)}^{2}} \end{array}\right.$$

The relative uncertainty of the temperature difference between the evaporation section and the condensation section is as follows:

(6) $$\frac{U_{\Delta T}}{\Delta T}=\sqrt{(\frac{U_{{\bar{T}}_{e}}}{{\bar{T}}_{e}}{)}^{2}+(\frac{U_{{\bar{T}}_{c}}}{{\bar{T}}_{c}}{)}^{2}}$$

In addition, the DC power supply input current and voltage accuracy is 1%. The uncertainty of the input power is less than 1.3%, and the relative uncertainties of the effective length and cross-sectional area of the UTVC are less than 0.1% and 2%, respectively. Then the uncertainties of the thermal resistance and equivalent thermal conductivity of the UTVC are calculated as follows:

(7) $$\left\{\begin{array}{l} \frac{U_{R}}{R}=\sqrt{(\frac{U_{P}}{P}{)}^{2}+(\frac{U_{\Delta T}}{\Delta T}{)}^{2}}\\ \frac{U_{K_{eff}}}{K_{eff}}=\sqrt{(\frac{U_{L_{eff}}}{L_{eff}}{)}^{2}+(\frac{U_{A_{C}}}{A_{c}}{)}^{2}+(\frac{U_{R}}{R}{)}^{2}} \end{array}\right.$$

In summary, the uncertainty of thermal resistance and ETC can be calculated according to Equations (5)–(7), and the uncertainty of the thermal resistance and the ETC of the UTVC is less than 4.71% and 5.12%, respectively.

## 3. Results and Discussion

### 3.1. Wettability of Wick

**Figure 5:**
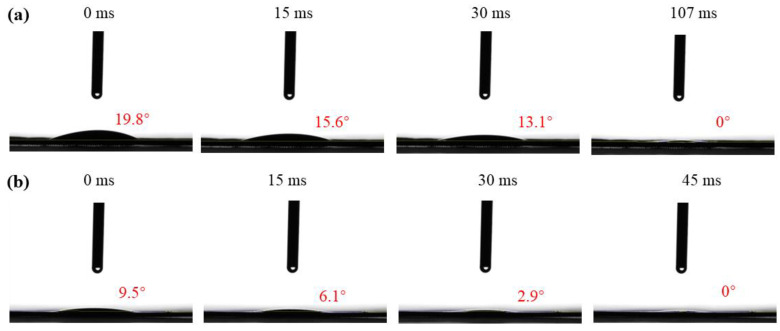
Wettability of wick. (**a**) With original SWM. (**b**) With oxidized SWM.

The contact angle test results of the wick before and after chemical treatment are shown in Figure 5a,b. It can be found that the initial contact angle of the wick with the original SWM is 19.8° at 0 ms, the contact angle becomes 15.6° at 15 ms, and the complete immersion time is 107 ms; the initial variation rate of the contact angle of the wick with the original SWM is 21% within the first 15 ms. By comparison, the initial contact angle of the wick with the oxidized SWM is 9.5° at 0 ms, the contact angle becomes 6.1° at 15 ms, and the complete immersion time is 45 ms; the initial variation rate of the contact angle of the wick with the oxidized SWM is 35.8% within the first 15 ms. The initial variation rate of the contact angle and complete immersion time of the wick with the oxidized SWM were significantly faster than that of the wick with the original SWM. This is because after the oxidation treatment, a layer of micro–nano structures grows on the copper surface, which greatly increases the surface roughness of the copper, thus significantly improving surface wettability.

### 3.2. Effect of Support Column Diameter

**Figure 6:**
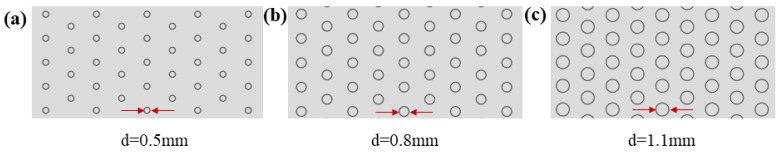
Support column structures of different diameters. (**a**) d = 0.5 mm. (**b**) d = 0.8 mm. (**c**) d = 1.1 mm.

Steam flow resistance is one of the most important factors affecting the thermal response performance of a UTVC. Due to the negative pressure inside the UTVC, the support column becomes an indispensable structure in the top shell. The larger the diameter of the support column, the stronger the pressure resistance of the UTVC, but the steam flow resistance also increases accordingly. Therefore, the influence of the support column diameter on the thermal response performance of the UTVC is explored in this paper, and the designed support column diameters of 0.5 mm, 0.8 mm, and 1.1 mm are shown in Figure 6.

**Figure 7:**
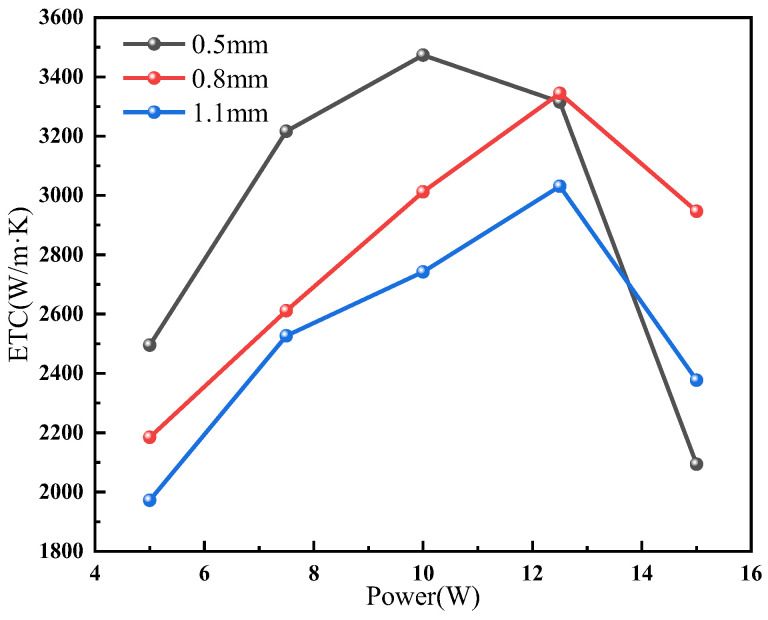
Heat transfer performance of UTVC with different support column diameters.

Figure 7 shows the heat transfer performance of the 30% FR UTVC with copper mesh (0 SWMs), and with different support column diameters of 0.5 mm, 0.8 mm, and 1.1 mm. It shows that the UTVC with 0.5 mm support column diameter has the highest ETC of 3473 W/(m·K), while the UTVCs with 0.8 mm and 1.1 mm support column diameters have the ETC of 3344 W/(m·K) and 3031 W/(m·K), respectively. The ETC of UTVC decreases as the support column diameter increases, and the ETC of a UTVC with a 0.5 mm support column is improved by 3.9% and 14.6% compared with that of 0.8 mm and 1.1 mm support column, respectively. The steam channel will increase as the diameter of the support column decreases, resulting in a decrease in the steam flow resistance of UTVC; this is beneficial for improving the evaporation rate of UTVC, as it increases the heat transfer performance of the UTVC. Therefore, the UTVC with the 0.5 mm support column diameter has the best heat transfer performance.

### 3.3. Effect of Different FRs

**Figure 8:**
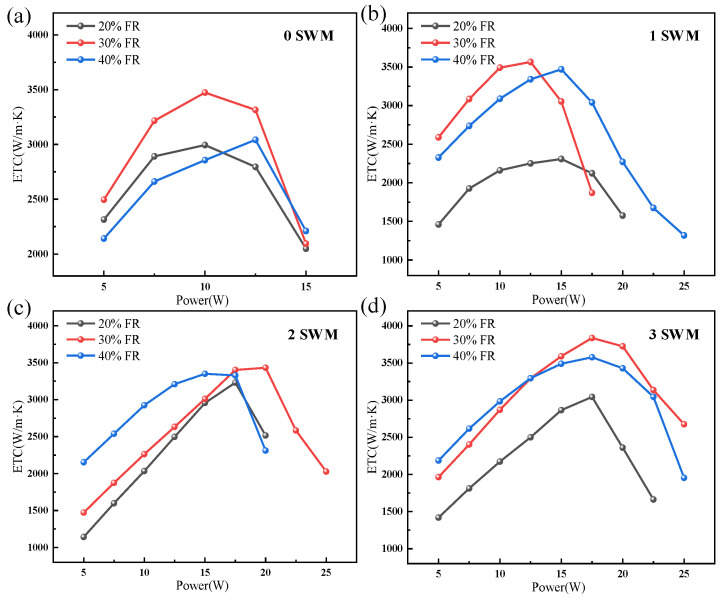
The impact of FRs on the ETC of UTVC: (**a**) 0 SWMs. (**b**) 1 SWM. (**c**) 2 SWMs; (**d**) 3 SWMs.

To explore the optimal FRs of the UTVC, under the experimental conditions where the support column diameter is 0.5 mm and the wick structures are 0 SWMs, 1 SWM, 2 SWMs, and 3 SWMs, respectively, the ETC of UTVCs with different FRs was experimentally investigated, as shown in Figure 8a–d. It can be found that for the different wick structures of the UTVCs, with the increase of heating power, the ETC of UTVCs increases first and then decreases. This is because steam generation in UTVCs increases with heating power, thus increasing the heat transfer efficiency of UTVCs under the condition of lower heating power. However, when the heating power is too high, due to the limited capacity of the wick, the large amount of steam generated cannot be sucked back to the evaporation section in time; therefore, the working fluid in the evaporation section cannot be replenished in a timely fashion, resulting in drying out or even failure of UTVCs. As a result, the ETC of UTVCs decreases when the power is too high.

Moreover, it also can be found that the effect of FRs on UTVCs with different numbers of SWMs (0 SWMs, 1 SWM, 2 SWMs, 3 SWMs) is consistent, the ETC of 30% FR is the highest, and the highest ETC is about 3473 W/(m·K), 3565 W/(m·K), 3350 W/(m·K) and 3837 W/(m·K), respectively. Compared with 20% FR, the ETC of 30% FR increased by 16%, 54.5%, 6.3%, and 26.1%, respectively, and compared with 40% FR, the ETC of 30% FR improved by 14.2%, 2.8%, 2.4%, and 7.3%, respectively. The reason is that when the FR is small, the returning liquid cannot meet the heat demand of the evaporation section and the evaporation section is in a burn-out state. However, when the FR is too high, the liquid level in the wick is relatively high, and the surface of the evaporation section is in the state of boiling and evaporation, resulting in greater vaporization resistance. At the same time, more liquid accumulates in the condensation section, which will cause liquid plugs during backflow and increases the backflow resistance. In general, under the conditions of four different structures of wicks, 30% FR is recommended to obtain better heat transfer performance.

### 3.4. Effect of Wick Structure

**Figure 9:**
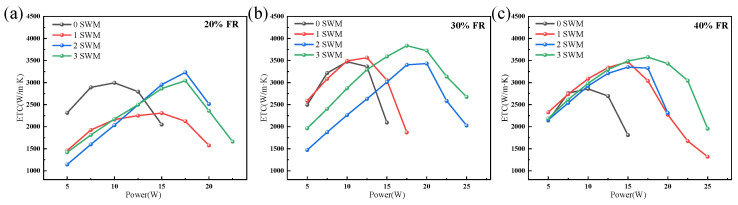
The ETC of UTVC with different SWMs: (**a**) 20% FR. (**b**) 30% FR. (**c**) 40% FR.

The capillary force of the wick has a critical influence on the heat transfer performance of the UTVC; the composite wicks mainly consist of two layers of copper, two layers of copper mesh+1 SWM, two layers of copper mesh+2 SWMs, and two layers of copper mesh+3 SWMs, and the influence of wick structures with SWM numbers of 0, 1, 2, and 3 (0 SWMs, 1 SWM, 2 SWMs, and 3 SWMs) on the heat transfer performance of UTVCs is explored in this section, as shown in Figure 9. It indicates that the ultimate power of UTVCs increases with the number of SWMs as a whole, and the peak value of ETC also moves in the direction of increasing power correspondingly. Specifically, the influence of SWM numbers on the ETC of the 20% FR UTVCs is shown in Figure 9a, when there is only copper mesh wick (0 SWMs), the ultimate power and maximum ETC of UTVC are about 16 W and 3000 W/(m·K), respectively. In comparison, the ultimate power and maximum ETC of the UTVC with 3 SWMs wick structure are approximately 23 W and 3000 W/(m·K), respectively. Additionally, under the working conditions of optimal 30% FR, the influence of different SWMs on UTVC is shown in Figure 9b. It shows that the ultimate powers of UTVCs with 0, 1, 2, and 3 SWMs are 15 W, 18 W, 26 W, and 26 W, respectively, and the corresponding ETC peaks are 3490 W/(m·K), 3565 W/(m·K), 3432 W/(m·K), and 3837 W/(m·K), respectively.

**Figure 10:**
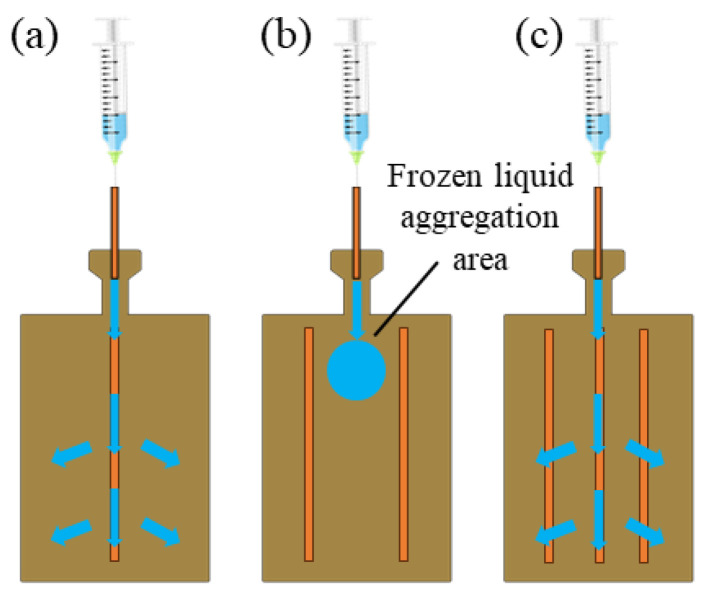
Schematic of SWM arrangement and liquid injection: (**a**) 1 SWM. (**b**) 2 SWMs. (**c**) 3 SWMs.

Moreover, the effect of the number of SWMs on the ETC of the 40% FR UTVC is shown in Figure 9c, the total trend of the effect of SWM number on the ETC is similar to that of 20% FR and 30% FR, the difference is that the ultimate power of 1 SWM exceeds that of 2 SWM, which is 25 W and 20 W, respectively. The reason is related to the arrangement of SWM and the injection and vacuum process of UTVC, the arrangement of 1 SWM and 3 SWM is shown in Figure 10a,c, the common feature is that they all have 1 SWM directly below the injection tube. When the working liquid is injected into the UTVC, the working liquid will rapidly diffuse to the bottom of the UTVC under the action of SWM capillarity. The UTVC with 2 SWMs is different from that having 1 SWM and 2 SWMs; as shown in Figure 10b, there is no SWM directly below the injection tube, while the other 2 SWMs are arranged on both sides of the injection tube, making it easy for the working fluid to remain below the injection tube. There is subsequently a frozen liquid accumulation area when the injection and vacuum process is carried out according to Section 2.1, resulting in an insufficient vacuum degree of UTVC with 2 SWMs. Moreover, the impact of vacuum degree on UTVC with 2 SWMs is more significant when FR is higher, since the frozen liquid aggregation area is larger, the corresponding insufficient vacuum degree leads to a decrease in the heat transfer performance of UTVC. Therefore, under the condition of 40% FR, the ultimate power of UTVC with 2 SWMs is lower than that of UTVC with 1 SWM.

To sum up, the maximum ETC of the UTVC increases with the number of SWMs, and at the same time, the ETC peak area of the UTVC moves towards higher power. This is because the SWM wick can significantly accelerate the return flow of liquid working fluid and ensure the stable operation of the gas–liquid phase change in the UTVC. However, since the SWM wick is arranged in the top shell, the volume of the vapor chamber of the UTVC will be compressed to a certain extent, thereby increasing the gas resistance. Therefore, the maximum ETC of the UTVC with different numbers of SWMs is not significantly improved.

### 3.5. Comparison with Reported Studies

To verify the superiority of the manufactured UTVC, the UTVC prepared in this paper is comprehensively compared with studies reported previously, which include thickness, ultimate power, and minimum thermal resistance, as listed in Table 2. Generally speaking, the ultimate power gradually decreases with the decrease in UTVC thickness; due to the reduction in thickness, the steam chamber is compressed, resulting in a serious loss of heat transfer performance of UTVC. Zhou et al. and Li et al. [15,18] enhanced the heat transfer performance of UTHPs by preparing different wick structures, and the results show that UTHPs have lower thermal resistance when the thickness of UTHPs is still above 1 mm and the ultimate power is below 24 W. Lim et al. [24] manufactured a UTVC with a grooved wick, indicating that there is almost no loss of cooling performance under antigravity conditions, but the UTVC has no advantages in thickness and ultimate power, as the thickness and ultimate power of the UTVC are 1.5 mm and 13 W, respectively. Huang et al. and Shi et al. [23,26] prepared UTVCs with thicknesses of 0.5 mm and 0.65 mm, but the ultimate power, which is 7.58 W and 13.7 W, respectively, was lost due to the decrease in thickness. Zhang et al. [35] manufactured a UTVC with a thickness of 0.32 mm, but the ultimate power was relatively low and the ultimate power was only 3 W. By comparison, the UTVC manufactured in this article has a superior thickness of 0.39 mm and an ultimate power of 26 W. This proves that the UTVC proposed in the article can maintain high ultimate power under reduced thickness through reasonable structural design according to Section 3.1, Section 3.2, Section 3.3 and Section 3.4.

Moreover, the comparison of the minimum thermal resistance and thickness of the UTVC proposed in this article with the reported literature is listed in Table 2. It can be seen that the thermal resistance of UTVC proposed by Zhou et al. [15], Li et al. [18], and that of the design proposed in this article is significantly lower than that of Lim et al. [24], Shi et al. [26], and Zhang et al. [35], the thermal resistance of UTVC of Zhou et al. [15], Li et al. [18], and this article is 0.092 °C/W, 0.05 °C/W, 0.56 °C/W, respectively. However, the thickness of the UTVC proposed by Zhou et al. [15] and Li et al. [18] is 1.5 mm and 1 mm, respectively. The thickness is obviously 2~3 times higher than the UTVC proposed in this article, once again confirming that the UTVC produced in this article takes into account both thickness and heat transfer performance (ultimate power and minimum thermal resistance), which is expected to achieve efficient heat dissipation in tiny electronics.

## 4. Conclusions

In this study, a 0.39 mm-thick UTVC with a composite wick is prepared; the composite wick contains two layers of copper mesh and multiple SWMs. Experimental research was conducted on the heat transfer performance of UTVCs with different support column diameters, FRs, and wick structures. The main conclusions are summarized as follows:

(1) The ETC with support column diameters of 0.5 mm, 0.8 mm, and 1.1 mm are compared under the condition of 30% FR and 0 SWMs. The UTVC with 0.5 mm support column diameter has the highest ETC of 3473 W/(m·K). By comparison, the UTVCs with 0.8 mm and 1.1 mm support column diameters have the ETC of 3344 W/(m·K) and 3031 W/(m·K), respectively. The ETC of UTVC with 0.5 mm support column is improved by 3.9% and 14.6% compared with that of 0.8 mm and 1.1 mm support column, the ETC decreases as the diameter of the support column increases.
(2) The effect of FRs on UTVCs with different numbers of SWMs (0 SWMs, 1 SWM, 2 SWMs, 3 SWMs) is consistent, the ETC of 30% FR is the highest, and the highest ETC is about 3473 W/(m·K), 3565 W/(m·K), 3350 W/(m·K) and 3837 W/(m·K), respectively. Compared with 20% FR, the ETC of 30% FR increased by 16%, 54.5%, 6.3%, and 26.1%, respectively, and compared with 40% FR, the ETC of 30% FR improved by 14.2%, 2.8%, 2.4%, and 7.3%, respectively.
(3) The increased number of SWMs can significantly improve the ultimate power of the UTVCs; under the condition of optimal 30% FR, the ultimate power of UTVCs with 0, 1, 2, and 3 SWMs are 15 W, 18 W, 26 W, and 26 W, respectively. an increase in the number of SWMs can slightly promote the maximum ETC of UTVCs, the maximum ETC of UTVCs with 0, 1, 2, and 3 SWMs are 3490 W/(m·K), 3565 W/(m·K), 3432 W/(m·K), and 3837 W/(m·K), respectively, indicating that the UTVC with the 3 SWMs has the highest ultimate power of 26 W. Moreover, the maximum ETC of UTVC with 0, 1, 2, and 3 SWMs shows slight improvement.

## Figures and Tables

**Table 1:** Design parameters of UTVC.

Structure/Parameters	Material/Dimensions (mm)
Shells and tube materials	Copper (C5191)
Working fluid	Deionized water
UTVC dimension (length × width × thickness)	58 × 82 × 0.4
Cavity dimension (length × width × thickness)	54 × 78 × 0.2
Wick structure	Copper mesh + SWM

**Table 2:** Comparison with other UTVC studies.

Literature	Thickness (mm)	Ultimate Power (W)	Minimum Thermal Resistance (°C/W)	Maximum ETC (W/(m·K))
Zhou et al. [15]	1.5	24	0.092	/
Li et al. [18]	1	14	0.05	/
Huang et al. [23]	0.5	7.58	/	25,200
Lim et al. [24]	1.5	13	5.45	/
Shi et al. [26]	0.65	13.7	1.15	800
Zhang et al. [35]	0.32	3	1.701	/
This work	0.39	26	0.56	3837

## Data Availability

Data underlying the results presented in this paper are available from the corresponding authors upon reasonable request.

## Nomenclature

Symbols	
$T_{1}$ and $T_{2}$ Temperatures of evaporation section, °C	$T_{3}$ and $T_{4}$ Temperatures of condensation section, °C
${\bar{T}}_{e}$ Average temperature of evaporating section, °C	${\bar{T}}_{c}$ Average temperature of condensing section, °C
ΔT Temperature difference, °C	R Total thermal resistance, °C/W
P Input power, W	$K_{eff}$ Equivalent thermal conductivity, W/(m·K)
$A_{c}$ Cross-sectional area, m^2^	L Effective length, m
$L_{e}$ Length of evaporating section, m	$L_{a}$ Length of adiabatic section, m
$L_{c}$ Length of condensing section, m	
Abbreviations	
UTVC Ultra-thin vapor chamber	SWM Spiral-woven mesh
FR Filling ratio	ETC Equivalent thermal conductivity
UTHP Ultra-thin heat pipe	SSGW Single arched sintered groove wick
BSGW Double-sided arched sintered groove wick	MGW Mesh groove wick
